# Earlier age at onset is associated with more severe sensory phenomena in drug-naive, comorbidity-free patients with obsessive-compulsive disorder

**DOI:** 10.3389/fpsyt.2026.1774594

**Published:** 2026-03-11

**Authors:** Makoto Kawahito, Keitaro Murayama, Hirofumi Tomiyama, Kenta Kato, Kou Matsukuma, Nami Nishida, Takuro Kamio, Kana Tsunoda, Kenta Sashikata, Mingi Kang, Ayaka Shuto, Shoma Tanaka, Tomohiro Nakao

**Affiliations:** 1 Department of Neuropsychiatry, Kyushu University, Fukuoka, Japan; 2 Department of Neuropsychiatry, Kyushu University Hospital, Fukuoka, Japan; 3 Fuculty of Education, Hokkaido University, Sapporo, Japan

**Keywords:** age at onset, autism spectrum traits, cross-sectional study, obsessive-compulsive disorder, sensory phenomena

## Abstract

**Introduction:**

Sensory phenomena (SP) are subjective experiences, such as feelings of discomfort or incompleteness, which often precede repetitive behaviors in patients with obsessive-compulsive disorder (OCD). Although previous studies have shown that SP are common in early-onset OCD, the relationship between age at onset and SP severity remains unclear.

**Methods:**

This cross-sectional study included 30 drug-naive patients with OCD, without comorbid psychiatric or medical/neurological disorders, and with at least one lifetime SP. SP severity was assessed using the University of São Paulo Sensory Phenomena Scale (USPSPS). Multiple regression analysis was conducted to examine the association between age at onset and SP severity, controlling for sex, autistic traits, and obsessive-compulsive symptom severity. Sensitivity analyses evaluated illness duration and anxiety and used a two-part analysis to address the floor at USPSPS = 0. Robustness was assessed using bias-corrected (BC) bootstrap 95% confidence intervals and influence diagnostics.

**Results:**

Earlier age at onset was associated with greater SP severity (*B* = −0.171, *p* = 0.007; BC bootstrap 95% CI −0.300 to −0.064). Sensitivity analyses, including models additionally adjusting for illness duration or anxiety, and influence diagnostics, supported the robustness of this association. In a two-part analysis, autistic traits were associated with the presence of current SP, whereas earlier onset was associated with greater SP severity.

**Discussion:**

Earlier onset of OCD was associated with more severe SP after adjustment for clinical covariates. These findings may be consistent with a neurodevelopmental contribution to SP severity in OCD. Further longitudinal and qualitative studies on SP are warranted.

## 1 Introduction

Obsessive-compulsive disorder (OCD) is a chronic psychiatric condition characterized by obsessions and compulsions that significantly impair daily functioning (1, 2). The disorder is relatively common, with a lifetime prevalence of 2.3–3.6% (3, 4). OCD shows heterogeneous symptom dimensions (e.g., contamination/washing, symmetry/ordering, aggression, and sexual/religious themes) (5). The etiology of OCD is multifactorial, involving genetic and environmental factors and neurobiological abnormalities, including dysfunction within cortico-striato-thalamo-cortical circuitry (6, 7). First-line treatments include pharmacotherapy and cognitive-behavioral therapy (CBT) (8). Selective serotonin reuptake inhibitors (SSRIs) are considered the pharmacological treatment of choice (9), and augmentation with atypical antipsychotics is recommended in treatment-resistant cases (10). Among psychotherapeutic options, exposure and response prevention (ERP) has demonstrated robust efficacy (11), especially when combined with pharmacotherapy (12).

Beyond symptom dimensions, sensory phenomena (SP) are increasingly recognized as clinically meaningful in OCD. SP are subjective experiences of physical or mental discomfort, tension, or incompleteness, which typically precede repetitive behaviors in OCD or tic disorders (13). These include a broad range of constructs, such as premonitory urges, “just-right” perceptions, and sensations of incompleteness (13). Conceptually, some SP are more sensory/urge-like (e.g., tactile or muscle-joint sensations), whereas others are more experiential/affective-cognitive (e.g., “not-just-right” experiences and feelings of incompleteness). The USP-SPS was designed to capture this broader SP construct (14). SP are considered particularly salient in the tic-related subtype of OCD (15, 16). SP are observed in approximately 65–72% of patients with OCD (14, 17, 18), and compulsive behaviors in these individuals are often not aimed at reducing anxiety or preventing harm, but at alleviating discomfort or fulfilling a sense of perfectionism (16). Notably, approximately 16% of patients with OCD report that SP are more distressing than their obsessions (17).

SP represent clinically relevant features in OCD that significantly influence symptom presentation, treatment response, and therapeutic strategies (15–17, 19, 20). Patients with SP tend to present with more severe symptoms in the dimensions of contamination/washing, symmetry/ordering, and aggression (15–17), although some studies have found no association between SP and overall OCD severity (15, 17), indicating the need for dimension-specific analyses. Overall severity refers to global OCD symptom burden (e.g., total Y-BOCS score), whereas dimension-specific severity refers to severity within symptom dimensions, typically assessed with dimensional instruments. Crucially, SP may show trait-like properties in nature rather than fluctuating with symptom severity (state-dependent) (21). For instance, Kano et al. (21) reported that in patients with Tourette’s syndrome, the severity of SP remained relatively stable over 4 years despite improvements in obsessive-compulsive symptoms. This stability aligns with emerging neurobiological evidence linking SP to specific neural dysfunctions. Specifically, SP have been linked to hyperactivity in the insular cortex, a region implicated in bodily awareness and interoception (22, 23), as well as dysfunction in sensorimotor networks (24). Sensory hypersensitivity is also prominent in autism spectrum disorder and may overlap phenomenologically with SP in OCD (25).

Regarding treatment, the presence of SP has been linked to variability in therapeutic outcomes. While some studies suggest that pharmacological efficacy is not significantly affected by the presence of tics (20), others indicate that patients with prominent feelings of incompleteness may respond poorly to standard therapeutic approaches, particularly cognitive therapy (19). In such cases, ERP focused on habituation to sensory urges may be particularly effective (19).

Previous studies have reported a higher prevalence of SP in early-onset OCD (14, 26). However, to our knowledge, no prior research has specifically examined the relationship between age at onset and the severity of SP. Therefore, the current study aimed to fill this gap by investigating the association between age at onset and SP severity in drug-naive patients with OCD with a lifetime history of SP. Examining severity provides a graded index of clinical impact (frequency, distress, and interference) and may refine neurodevelopmental interpretations beyond presence/absence. We hypothesized that an earlier age at onset would be associated with greater SP severity. This hypothesis is based on previous findings that SP are more frequently observed in early-onset OCD (14, 26) and on the notion that early-onset OCD may reflect a neurodevelopmentally distinct subtype potentially characterized by increased vulnerability to sensory-driven compulsivity (24, 27, 28).

## 2 Materials and methods

### 2.1 Participants and procedure

**Figure 1:**
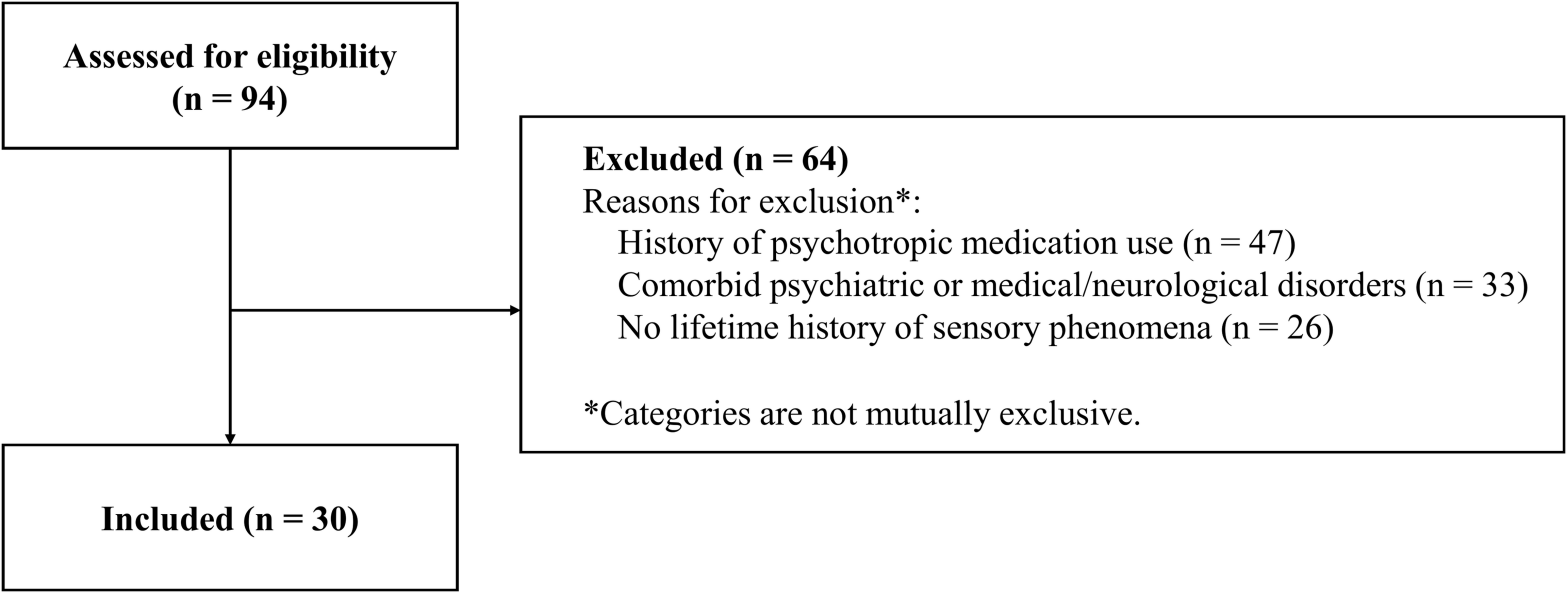
Flowchart of participant selection. A total of 94 patients with obsessive-compulsive disorder (OCD) were screened, and 64 were excluded due to a history of psychotropic medication use (n = 47), comorbid psychiatric or medical/neurological disorders (n = 33), or no lifetime history of sensory phenomena (n = 26). Detailed breakdowns of comorbidities and medication history are provided in **Supplementary Tables S1**, **S2**.

Patients were recruited from the Department of Neuropsychiatry, Kyushu University Hospital, between February 2016 and April 2025. During this period, a total of 94 individuals were diagnosed with OCD according to DSM-5 criteria and expressed willingness to undergo eligibility screening. As detailed in the study flowchart (**Figure 1**), of the 94 patients screened, 64 were excluded primarily due to lifetime psychotropic medication use, comorbidities, or no history of SP (**Supplementary Tables S1**, **S2**). Comorbid psychiatric disorders included tic disorders. Therefore, no included participant met DSM-5 criteria for a tic disorder. A total of 30 patients met all eligibility criteria and were included in the final analysis. Participants were eligible for inclusion if they met the following criteria: (1) age at assessment between 15 and 65 years, (2) drug-naive, defined as having no lifetime history of psychotropic medication use, (3) absence of comorbid psychiatric disorders or medical/neurological conditions that could affect sensory assessment, and (4) a lifetime history of at least one SP (either current or previous; see definitions below). During eligibility screening, the USP-SPS checklist was administered to all screened patients to determine the presence of lifetime SP. Patients who did not endorse any lifetime SP were excluded from the present study.

All diagnoses were made by board-certified psychiatrists using the Structured Clinical Interview for DSM-5 (SCID-5), following standard training protocols. The study was approved by the Institutional Review Board of Kyushu University (Approval No. 22294-02). Written informed consent was obtained from all participants after a thorough explanation of the study objectives and procedures. For participants who were minors, consent was also obtained from a parent, as required.

### 2.2 Assessment tools

#### 2.2.1 Structured clinical interview for DSM-5

The SCID-5 is a widely used structured interview for diagnosing psychiatric disorders based on DSM-5 criteria (29). It was used to assess OCD and to exclude comorbid psychiatric disorders. Notably, the SCID-5 does not provide a structured assessment for ASD; therefore, autistic traits were additionally evaluated using the AQ. Age at onset was defined as the age at which OCD symptoms first caused clinically significant distress or functional impairment, as determined during the clinical interview. Age at onset was retrospectively assessed based on the patient’s report.

#### 2.2.2 University of São Paulo sensory phenomena scale

The USP-SPS is a semi-structured interview developed to assess the presence and severity of SP (14). The instrument consists of two parts: a checklist and severity scale. The checklist covers five major categories of SP—physical sensations, “just-right” perceptions, feeling of incompleteness, energy release, and “urge-only” phenomena—with items assessing both current and past SP experiences and the age of their onset. Current SP was defined as symptoms present within the past month. Previous SP was defined as symptoms experienced at any point in the patient’s lifetime prior to the past month. The severity scale comprises three items evaluating frequency, distress, and interference in daily life, each rated on a 0–5 scale. The total severity score ranges from 0–15 (14).

In the present study, participants were required to endorse at least one SP on the checklist (current or previous). Thus, a USP-SPS severity score of 0 indicated no SP during the past month in individuals with a lifetime history of SP. The Japanese version of the USP-SPS was used, which was developed through a rigorous translation and back-translation procedure, as described by Kano et al. (30). The scale was administered in a semi-structured interview format by trained psychiatrists.

#### 2.2.3 Yale-brown obsessive-compulsive scale

The Y-BOCS is the most widely used clinician-rated measure of OCD severity. It assesses five severity parameters for both obsessions and compulsions: time, interference, distress, resistance, and control. Each item is rated on a 0–4 scale, with a total score ranging from 0–40 (31). The Y-BOCS is a reliable and valid measure for research on OCD (32).

#### 2.2.4 Autism-spectrum quotient

Autistic traits are increasingly recognized as relevant to OCD phenomenology. Sensory hypersensitivity is a core feature of autism spectrum disorder (ASD) and overlaps phenomenologically with SP in OCD (25). Given this overlap, it is crucial to disentangle whether SP severity is driven primarily by OCD pathology or by underlying autistic traits. However, categorical diagnostic assessments may not capture the full continuum of these traits. Therefore, we additionally evaluated autistic traits using the AQ to control for this potential confounder. The AQ is a self-report questionnaire designed to quantify autistic traits. It comprises 50 items across five domains: social skills, attention switching, attention to detail, imagination, and communication. Total scores range from 0–50, with higher scores indicating greater autistic traits (33). The AQ is commonly used as a screening measure to indicate whether further assessment may be warranted; in the present study, it was used dimensionally as a covariate. The Japanese version has demonstrated adequate reliability (34).

#### 2.2.5 Hamilton anxiety rating scale

The HAM-A is a clinician-administered scale used to assess the severity of anxiety symptoms across 14 items, encompassing both psychological and somatic components. Each item is rated from 0–4, yielding a total score from 0–56 (35). In this study, the Japanese version was administered by trained raters (36, 37).

### 2.3 Statistical analysis

A multiple linear regression analysis was conducted to examine the association between age at onset and SP severity, adjusting for sex, autistic traits (AQ), and obsessive-compulsive symptom severity (Y-BOCS). Unstandardized regression coefficients (*B*) were calculated. All variables were entered simultaneously. The dependent variable was the total USP-SPS severity score, indexing the severity of SP during the past month among patients with a lifetime history of SP (range 0–15). Statistical significance was set at a two-tailed *p* value < 0.05. JMP Pro version 18.2.1 (SAS Institute Inc., Cary, NC, USA) was used for all analyses.

Prior to regression analysis, statistical assumptions were verified. Normality of residuals was assessed using the Shapiro–Wilk test and the Anderson–Darling test, along with visual inspection of normal Q–Q plots. Linearity and homoscedasticity were assessed by examining residual-versus-fitted plots. Multicollinearity was evaluated using variance inflation factors (VIFs) (all < 2.0).

The AQ and Y-BOCS scores were included as covariates based on prior studies indicating their associations with SP (17, 38, 39). Sex was included as a covariate to adjust for established epidemiological differences in OCD prevalence and symptom presentation. Illness duration and anxiety symptom severity (HAM-A) were not included in the primary model to preserve degrees of freedom in this small sample. Illness duration was calculated as the interval between age at onset and age at assessment, expressed in months. Because age at assessment is mathematically determined by age at onset plus illness duration, we did not include age at assessment in the primary model to avoid multicollinearity and interpretational ambiguity. Instead, we evaluated potential age-related confounding by refitting the primary model with illness duration added as a sensitivity analysis. HAM-A was examined in a separate sensitivity analysis by adding it to the primary model as additional covariate.

To address the floor mass at USP-SPS = 0 (current SP absence), we additionally conducted a two-part analysis. First, a binary logistic regression model was fitted to examine factors associated with the presence of current SP (USP-SPS > 0 vs USP-SPS = 0), using age at onset, sex, AQ, and Y-BOCS as predictors. Results are reported as odds ratios (ORs) with 95% confidence intervals. Second, among participants with USP-SPS > 0, a multiple linear regression model was fitted to examine the association between age at onset and SP severity using the same covariates.

To assess robustness under potential deviations from parametric assumptions and the small sample size, we conducted a nonparametric bootstrap (5,000 resamples) to obtain bias-corrected (BC) 95% confidence intervals for the regression coefficient of age at onset in the primary linear regression model. Influential observations were evaluated using Cook’s distance and leverage (hat values). Observations were flagged as potentially influential if Cook’s distance exceeded 4/n and/or leverage exceeded 2p/n (where n is the sample size and p is the number of model parameters including the intercept [p = 5 in the primary model]). As a sensitivity analysis, we refitted the model after excluding flagged observations.

## 3 Results

### 3.1 Participant characteristics

This study included 30 drug-naive patients with OCD who had a lifetime history of at least one SP, and had no comorbid psychiatric or medical/neurological disorders. The mean age of the participants was 31.7 years (standard deviation [SD] = 14.6), and 21 (70%) were female. The mean age at OCD onset was 22.4 years (SD = 13.3), and the mean illness duration was 109 months (SD = 127). The mean total Y-BOCS score was 22.7 (SD = 6.1), with a mean score of 11.2 (SD = 3.7) for obsessions and 11.5 (SD = 2.9) for compulsions. The mean USP-SPS score was 6.0 (SD = 4.7). Seven participants (23.3%) had a USP-SPS severity score of 0 (no SP during the past month despite a lifetime history of SP). The mean AQ score was 24.3 (SD = 7.2), and the mean HAM-A score was 6.0 (SD = 5.7). Details are presented in **Table 1**.

**Table 1:** Participant characteristics [Mean (Standard Deviation)].

Characteristic	Total sample (n = 30)
Sex, female, n (%)	21 (70.0)
Age, years	31.7 (14.6)
Age at OCD onset, years	22.4 (13.3)
AQ	24.3 (7.2)
Y-BOCS total	22.7 (6.1)
Y-BOCS obsession	11.2 (3.7)
Y-BOCS compulsion	11.5 (2.9)
Duration of illness, months	109 (127)
USP-SPS severity (past month)	6.0 (4.7)
USP-SPS = 0 (past month), n (%)	7 (23.3)
HAM-A	6.0 (5.7)

All participants were drug-naive and had no comorbid psychiatric or medical/neurological disorders. USP-SPS = 0 indicates no sensory phenomena in the past month (not the absence of lifetime sensory phenomena). No missing data. Abbreviations: AQ, Autism-Spectrum Quotient; Y-BOCS, Yale-Brown Obsessive Compulsive Scale; USP-SPS, University of São Paulo Sensory Phenomena Scale; HAM-A, Hamilton Anxiety Rating Scale.

Regarding the distribution of SP subtypes (**Table 2**), the most frequently reported current phenomena were feelings of incompleteness (56.7%), tactile physical sensations (40%), and “urge-only” phenomena (20%). Other notable current symptoms included energy release (16.7%) and auditory or visual “just-right” perceptions (13.3% each), while muscle-joint sensations (3.3%) and tactile “just-right” perceptions (10%) were less common. Detailed frequencies of all SP subtypes are provided in **Table 2**. Because multiple SP subtypes could co-occur within individuals, percentages do not sum to 100%.

**Table 2:** Frequency of SP subtype (n = 30) [Number (Percentage)].

SP subtype	Previous (lifetime, not past month)	Current (past month)
A. Physical sensations		
A1. Tactile	9 (30.0%)	12 (40.0%)
A2. Muscle-joint	2 (6.7%)	1 (3.3%)
B. “Just-right” perceptions		
B1. Visual	4 (13.3%)	3 (10.0%)
B2. Auditory	4 (13.3%)	4 (13.3%)
B3. Tactile	2 (6.7%)	3 (10.0%)
C. Feeling of incompleteness	1 (3.3%)	17 (56.7%)
D. Energy release	3 (10.0%)	5 (16.7%)
E. “Urge-only” phenomena	2 (6.7%)	6 (20.0%)

All participants had a lifetime history of SP. Current SP indicates symptoms present within the past month; otherwise, symptoms were coded as Previous SP if endorsed for any time before the past month. Percentages are based on n = 30 and do not sum to 100% because participants could endorse multiple SP subtypes. Abbreviation: SP, sensory phenomena.

### 3.2 Primary regression analysis

**Figure 2:**
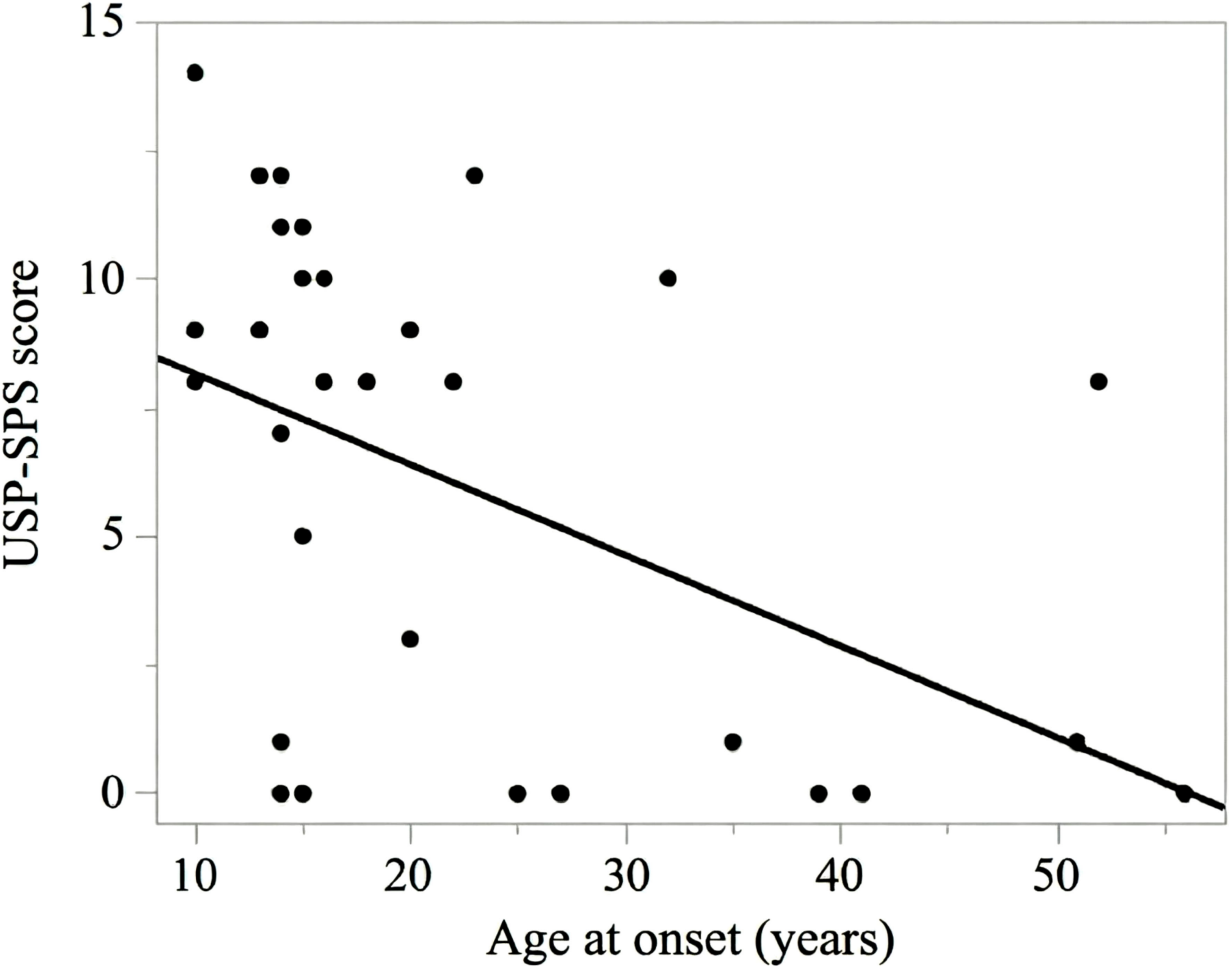
Scatterplot of the association between age at onset and sensory phenomena severity. Each dot represents one participant with OCD and a lifetime history of SP (n = 30). The x-axis represents age at OCD onset (years), and the y-axis represents the total USP-SPS severity score (past month; range 0–15). The solid line represents the fitted unadjusted simple linear regression line for visualization; primary inferences are based on the adjusted models reported in the main text. Abbreviations: SP, sensory phenomena; OCD, obsessive-compulsive disorder; USP-SPS, University of São Paulo Sensory Phenomena Scale.

In the multiple regression model, age at onset was significantly associated with SP severity (*B* = −0.171, *p* = 0.007), indicating that earlier onset was associated with greater SP severity. The association remained robust in BC bootstrap resampling (BC bootstrap 95% CI: −0.300 to −0.064; 5,000 resamples). The bivariate (unadjusted) relationship between age at onset and USP-SPS severity scores is shown in **Figure 2**.

AQ was also positively associated with SP severity (*B* = 0.267, *p* = 0.031), whereas sex and Y-BOCS were not significant predictors. The overall model was statistically significant (*F*(4, 25) = 4.12, *p* = 0.011; adjusted *R²* = 0.301). Detailed regression results are presented in **Table 3**. Model diagnostics did not indicate major violations of assumptions (residual normality: Shapiro–Wilk *W* = 0.972, *p* = 0.607; Anderson–Darling *A²* = 0.315, *p* = 0.546).

**Table 3:** Results of multiple regression analysis predicting USP-SPS severity (past month).

Predictor	*B* (unstandardized)	SE	*t*	*p*-value	BC bootstrap 95% CI for *B*
Intercept	6.267	4.195	1.49	0.148	[–2.100, 14.430]
Age at onset	–0.171	0.058	–2.93	0.007**	[–0.300, –0.064]
Sex (female vs male)	1.384	0.866	1.60	0.123	[–0.100, 3.150]
AQ total	0.267	0.117	2.29	0.031*	[0.045, 0.463]
Y-BOCS total	–0.155	0.131	–1.18	0.248	[–0.414, 0.077]

N = 30. Adjusted *R²* = 0.301. Model df = 4, residual df = 25. The coefficient for sex represents the estimated change from male to female. Bias-corrected (BC) bootstrap 95% confidence intervals are reported for the unstandardized regression coefficients (*B*) and were obtained using 5,000 nonparametric resamples. Abbreviations: SE, standard error; AQ, Autism-Spectrum Quotient; Y-BOCS, Yale-Brown Obsessive Compulsive Scale. *p < 0.05, **p < 0.01.

Influence diagnostics identified one observation exceeding the prespecified Cook’s distance threshold (Cook’s D > 4/n; threshold = 0.133 for n = 30), while none exceeded the leverage threshold (h > 2p/n = 0.333; *p* = number of parameters including the intercept = 5). As a prespecified sensitivity analysis, the model was refitted after excluding the flagged observation (n = 29). The inverse association between age at onset and SP severity remained significant (*B* = −0.171, *p* = 0.009), and AQ remained significant (*B* = 0.267, *p* = 0.037).

### 3.3 Sensitivity analyses

To examine the robustness of the association between age at onset and SP severity, sensitivity analyses were conducted. First, a simple linear regression using only age at onset as a predictor revealed a significant negative association (*B* = −0.176, *p* = 0.005; adjusted *R*² = 0.226). The association remained significant when adding illness duration (*B* = −0.168, *p* = 0.011) or HAM-A (*B* = −0.172, *p* = 0.008) separately, while these added covariates were not significant (illness duration *p* = 0.796; HAM-A *p* = 0.887). Detailed results are provided in **Table 4**.

**Table 4:** Sensitivity analyses for the association between age at onset and SP severity.

Model	Covariates	*B* (unstandardized)	SE	*t*	*p*-value	Adjusted *R²*
Simple linear regression	None	–0.176	0.057	–3.07	0.005 **	0.226
Primary multiple regression	Sex, AQ, Y-BOCS	–0.171	0.058	–2.93	0.007 **	0.301
Primary model + illness duration (months)	+ illness duration	–0.168	0.061	–2.77	0.011 *	0.274
Primary model + HAM-A	+ HAM-A	–0.172	0.060	–2.87	0.008 **	0.272

All models: n = 30. Coefficients (*B*) represent the association between age at onset (years) and USP-SPS severity (past month). Adjusted *R²* indicates the adjusted coefficient of determination for each model. Abbreviations: SP, sensory phenomena; SE, standard error; AQ, Autism-Spectrum Quotient; Y-BOCS, Yale-Brown Obsessive Compulsive Scale; HAM-A, Hamilton Anxiety Rating Scale. *p < 0.05, **p < 0.01.

### 3.4 Two-part analysis for the floor mass at USP-SPS = 0

Given the floor at USP-SPS = 0 (indicating no SP during the past month), we performed an additional two-part analysis (**Supplementary Table S3**). In the binary logistic regression model predicting the presence of current SP (USP-SPS > 0 vs USP-SPS = 0), AQ was significantly associated with current SP (OR = 1.24 per 1-point increase; 95% CI 1.04–1.57; *p* = 0.012), whereas age at onset showed a trend-level association (OR = 0.93 per 1-year increase; 95% CI 0.84–1.01; *p* = 0.076).

Among participants with USP-SPS > 0 (n = 23), age at onset was negatively associated with SP severity (*B* = −0.144, *p* = 0.0497). After excluding one influential observation identified by Cook’s distance (n = 22), the association remained significant (*B* = −0.134, *p* = 0.030). Full model outputs are reported in **Supplementary Table S3**. Given the small number of participants with USP-SPS = 0, the logistic component (Part 1) should be interpreted cautiously as exploratory and potentially imprecise.

## 4 Discussion

This study demonstrated that an earlier age at onset was significantly associated with greater SP severity in patients with OCD who had a lifetime history of SP. This extends prior work showing that SP are more common in early-onset OCD by suggesting a graded relationship between onset age and the clinical impact of SP (frequency, distress, and interference), even after adjustment for overall OCD severity and autistic traits. This finding may be consistent with the possibility that neurodevelopmental vulnerability contributes to the formation and persistence of SP. In practical terms, the unstandardized coefficient indicates that a 10-year earlier onset of OCD is associated with approximately a 1.7-point increase in USP-SPS severity score. This relationship remained even after controlling for potential confounders in the primary multiple regression model (sex, AQ, and Y-BOCS), and it also remained significant when illness duration or HAM-A was added separately in sensitivity analyses. These findings align with those of previous studies indicating that SP are more frequently observed in early-onset OCD (14, 26). However, the explanatory power of the primary regression model was modest to moderate (adjusted *R*² = 0.301), suggesting that additional unmeasured factors also contribute to SP severity.

Robustness analyses further supported the main finding. The bias-corrected bootstrap confidence interval for the age at onset coefficient excluded zero (BC 95% CI: −0.300 to −0.064), suggesting that the association is unlikely to be solely driven by parametric assumptions in this small sample. The confidence interval indicates a statistically reliable, but clinically modest effect size, underscoring the need for replication in larger cohorts. Influence diagnostics also suggested that the finding was not driven by a small number of influential observations: one observation exceeded the prespecified Cook’s distance threshold (Cook’s D > 4/n), and refitting the model after excluding this observation (n = 29) yielded a similar estimate and significance level for age at onset.

Early-onset OCD has been associated with neurodevelopmental vulnerabilities, such as increased thalamic volume (28) and demonstrated asymmetry in the thalamus and globus pallidus in pediatric patients (27). Neuroimaging studies have also suggested that patients with OCD and SP have greater volumes in the basal ganglia, including the thalamus and globus pallidus, than healthy controls, and exhibit dysfunction in sensorimotor networks (24). Furthermore, studies of cortical development trajectories have shown that cortical thickness in the insula, particularly the posterior region, reaches its peak later, around the age of 18 years (40). Accordingly, in individuals with early-onset OCD, obsessive-compulsive behaviors may develop during a critical window of insular maturation, potentially contributing to more severe SP. Taken together, these findings raise the possibility that SP may serve as a marker of a neurodevelopmental subtype of OCD. However, because the present study is cross-sectional and age at onset was assessed retrospectively, these neurodevelopmental interpretations are hypothesis generating. Longitudinal studies with prospective onset characterization and repeated SP assessments are needed.

Clinically, individuals with OCD and SP are often motivated to perform compulsions to alleviate a sense of incompleteness or uncomfortable bodily sensations (16). In settings where SP are not routinely emphasized, earlier-onset OCD may prompt clinicians to proactively inquire about SP, while recognizing that age at onset explains only a modest proportion of variance in SP severity and should not be used as a substitute for SP assessment. Specialized ERP strategies, which may be more effective than cognitive therapy in patients with SP, should be considered (19). Standard ERP targeting fear-based obsessions may be less effective for the “just-right” sensations characteristic of SP. Our findings suggest that, particularly in patients with prominent SP, treatment strategies should be personalized to include sensory-driven urges and experiences of incompleteness, rather than solely focusing on anxiety reduction (19). Evidence on pharmacological strategies specifically targeting SP in OCD remains limited. Much of the available treatment literature addresses tic-related OCD, a subgroup in which SP are frequent, and suggests that antipsychotic augmentation of SSRIs may be beneficial in some patients, particularly those with comorbid tics (41, 42). Therefore, any pharmacological implications for SP should be considered preliminary and require studies that directly phenotype SP severity and subtype.

Because USP-SPS severity had a floor at zero, we conducted a two-part analysis (**Supplementary Table S3**) to distinguish correlates of current SP presence from correlates of severity among those with current SP. Autistic traits were associated with current SP presence, whereas earlier age at onset was associated with greater SP severity, suggesting potentially distinct correlates for SP presence and severity. Although AQ was positively associated with SP severity in the primary model, it was not associated with severity in Part 2; therefore, any association between autistic traits and SP severity should be interpreted cautiously and requires replication. Prior work links autistic traits to atypical sensory processing both in ASD and in non-clinical populations (25, 38, 43), which may contribute to the experience of SP in OCD. It is also important to distinguish related but nonequivalent constructs. Sensory sensitivity/processing differences, often studied in ASD, typically refer to heightened or atypical responsiveness to external sensory input. In contrast, SP in OCD as measured by the USP−SPS emphasize subjective urges or “not-just-right”/incompleteness experiences that precede or accompany repetitive behaviors. These constructs may overlap at a phenomenological level but are not interchangeable.

This study has several limitations. First, the sample size was small (n = 30), reflecting stringent criteria to obtain a drug-naive, comorbidity-free sample, which limited statistical power and precision. Given the sample size relative to the number of predictors, estimates may be unstable; therefore, we used BC bootstrap confidence intervals, prespecified sensitivity analyses (univariate and single-covariate addition models), influence diagnostics, and a two-part analysis to address the floor at USP-SPS = 0, which supported the main finding. Second, age at onset was skewed toward younger ages, limiting generalizability to late-onset OCD. In addition, age at onset was retrospectively assessed, which may introduce recall bias and measurement variability. Third, OCD symptom dimensions were not systematically assessed, precluding examination of dimension-specific effects. The sample had on average moderate OCD severity (mean Y-BOCS = 22.7), which may limit generalizability to more severe clinical samples. Fourth, SP are heterogeneous, ranging from bodily/urge-like sensations to experiential phenomena such as “not-just-right” experiences and feelings of incompleteness. Although the USP−SPS was designed to capture this broad SP construct (14), the severity scale yields a single total score and does not provide subtype-specific severity indices. Therefore, and given our small sample size, we analyzed total USP−SPS severity and could not examine whether age at onset is differentially associated with specific SP categories. Future studies with larger samples should assess subtype-specific severity and test differential associations across SP subtypes.

Fifth, because all participants had a lifetime history of SP, our findings address variability in SP severity among SP-positive patients rather than the presence versus absence of lifetime SP. Finally, the cross-sectional design precludes causal inference.

## 5 Conclusion

To our knowledge, this study provides novel evidence that age at onset is associated with the severity of SP among patients with OCD who have a lifetime history of SP. Multiple regression analyses revealed that an earlier age at onset was consistently associated with greater SP severity, even after controlling for potential confounders. This association remained robust across bootstrap resampling and sensitivity analyses. These findings may be consistent with the hypothesis that SP reflect neurodevelopmental vulnerability in OCD among patients who experience SP, although this interpretation remains hypothetical given the cross-sectional design. In an additional two-part analysis, autistic traits were associated with current SP presence, whereas earlier onset was associated with greater SP severity among those with current SP. Clinically, assessing age at onset may help inform individualized treatment strategies for patients with OCD who experience SP, particularly those with prominent sensory-driven compulsions. Future research should employ longitudinal designs in larger and more heterogeneous samples and explore the qualitative aspects of SP to further elucidate their developmental and clinical significance.

## Data Availability

The raw data supporting the conclusions of this article will be made available by the authors, without undue reservation.

## References

[1] American Psychiatric Association . Diagnostic and Statistical Manual of Mental Disorders. 5th ed. Washington, DC: American Psychiatric Publishing (2013).

[2] Coluccia A Fagiolini A Ferretti F Pozza A Costoloni G Bolognesi S . Adult obsessive-compulsive disorder and quality of life outcomes: A systematic review and meta-analysis. Asian J Psychiatr. (2016) 22:41–52. doi: 10.1016/j.ajp.2016.02.001, PMID: 27520893

[3] Ruscio AM Stein DJ Chiu WT Kessler RC . The epidemiology of obsessive-compulsive disorder in the National Comorbidity Survey Replication. Mol Psychiatry. (2010) 15:53–63. doi: 10.1038/mp.2008.94, PMID: 18725912 PMC2797569

[4] Subramaniam M Abdin E Vaingankar J Shafie S Chang S Seow E . Obsessive-compulsive disorder in Singapore: prevalence, comorbidity, quality of life and social support. Ann Acad Med Singap. (2020) 49:15–25. doi: 10.47102/annals-acadmedsg.2019185 32200393

[5] Rosario-Campos MC Miguel EC Quatrano S Chacon P Ferrao Y Findley D . The Dimensional Yale–Brown Obsessive-Compulsive Scale (DY-BOCS): an instrument for assessing obsessive-compulsive symptom dimensions. Mol Psychiatry. (2006) 11:495–504. doi: 10.1038/sj.mp.4001798, PMID: 16432526

[6] Strom NI Gerring ZF Galimberti M Yu D Halvorsen MW Abdellaoui A . Genome-wide analyses identify 30 loci associated with obsessive-compulsive disorder. Nat Genet. (2025) 57:1389–401. doi: 10.1038/s41588-025-02189-z, PMID: 40360802 PMC12165847

[7] Milad MR Rauch SL . Obsessive-compulsive disorder: beyond segregated cortico-striatal pathways. Trends Cognit Sci. (2012) 16:43–51. doi: 10.1016/j.tics.2011.11.003, PMID: 22138231 PMC4955838

[8] Singh A Anjankar VP Sapkale B . Obsessive-compulsive disorder (OCD): A comprehensive review of diagnosis, comorbidities, and treatment approaches. Cureus. (2023) 15:e48960. doi: 10.7759/cureus.48960, PMID: 38111433 PMC10726089

[9] Soomro GM Altman DG Rajagopal S Oakley-Browne M . Selective serotonin re-uptake inhibitors (SSRIs) versus placebo for obsessive compulsive disorder (OCD). Cochrane Database Syst Rev. (2008) 2008:CD001765. doi: 10.1002/14651858.CD001765.pub3, PMID: 18253995 PMC7025764

[10] Albert U Marazziti D Di Salvo G Solia F Rosso G Maina G . A systematic review of evidence-based treatment strategies for obsessive-compulsive disorder resistant to first-line pharmacotherapy. Curr Med Chem. (2018) 25:5647–61. doi: 10.2174/0929867325666171222163645, PMID: 29278206

[11] Reid JE Laws KR Drummond L Vismara M Grancini B Mpavaenda D . Cognitive behavioural therapy with exposure and response prevention in the treatment of obsessive-compulsive disorder: A systematic review and meta-analysis of randomised controlled trials. Compr Psychiatry. (2021) 106:152223. doi: 10.1016/j.comppsych.2021.152223, PMID: 33618297

[12] O’Connor K Todorov C Robillard S Borgeat F Brault M . Cognitive-behaviour therapy and medication in the treatment of obsessive-compulsive disorder: a controlled study. Can J Psychiatry. (1999) 44:64–71. doi: 10.1177/070674379904400108, PMID: 10076743

[13] Prado HS Rosário MC Lee J Hounie AG Shavitt RG Miguel EC . Sensory phenomena in obsessive-compulsive disorder and tic disorders: a review of the literature. CNS Spectr. (2008) 13:425–32. doi: 10.1017/S1092852900016606, PMID: 18496480

[14] Rosario MC Prado HS Borcato S Diniz JB Shavitt RG Hounie AG . Validation of the University of São Paulo Sensory Phenomena Scale: initial psychometric properties. CNS Spectr. (2009) 14:315–23. doi: 10.1017/s1092852900020319, PMID: 19668122

[15] de Vries FE Cath DC Hoogendoorn AW van Oppen P Glas G Veltman DJ . Tic-related versus tic-free obsessive-compulsive disorder: clinical picture and 2-year natural course. J Clin Psychiatry. (2016) 77:e1240–7. doi: 10.4088/JCP.14m09736, PMID: 27631146

[16] Brander G Kuja-Halkola R Rosenqvist MA Rück C Serlachius E Fernández de la Cruz L . A population-based family clustering study of tic-related obsessive-compulsive disorder. Mol Psychiatry. (2021) 26:1224–33. doi: 10.1038/s41380-019-0532-z, PMID: 31616041 PMC7985024

[17] Ferrão YA Shavitt RG Prado H Fontenelle LF Malavazzi DM de Mathis MA . Sensory phenomena associated with repetitive behaviors in obsessive-compulsive disorder: an exploratory study of 1001 patients. Psychiatry Res. (2012) 197:253–8. doi: 10.1016/j.psychres.2011.09.017, PMID: 22361443

[18] Gomes de Alvarenga P de Mathis MA Dominguez Alves AC do Rosário MC Fossaluza V Hounie AG . Clinical features of tic-related obsessive-compulsive disorder: results from a large multicenter study. CNS Spectr. (2012) 17:87–93. doi: 10.1017/S1092852912000491, PMID: 22789066

[19] Summerfeldt LJ . Understanding and treating incompleteness in obsessive-compulsive disorder. J Clin Psychol. (2004) 60:1155–68. doi: 10.1002/jclp.20080, PMID: 15389620

[20] Husted DS Shapira NA Murphy TK Mann GD Ward HE Goodman WK . Effect of comorbid tics on a clinically meaningful response to 8-week open-label trial of fluoxetine in obsessive compulsive disorder. J Psychiatr Res. (2007) 41:332–7. doi: 10.1016/j.jpsychires.2006.05.007, PMID: 16860338

[21] Kano Y Fujio M Kaji N Matsuda N Nonaka M Kono T . Changes in sensory phenomena, tics, obsessive-compulsive symptoms, and global functioning of Tourette syndrome: a follow-up after four years. Front Psychiatry. (2020) 11:619. doi: 10.3389/fpsyt.2020.00619, PMID: 32695033 PMC7338586

[22] Tinaz S Malone P Hallett M Horovitz SG . Role of the right dorsal anterior insula in the urge to tic in Tourette syndrome. Mov Disord. (2015) 30:1190–7. doi: 10.1002/mds.26230, PMID: 25855089 PMC5088605

[23] Brown C Shahab R Collins K Fleysher L Goodman WK Burdick KE . Functional neural mechanisms of sensory phenomena in obsessive-compulsive disorder. J Psychiatr Res. (2019) 109:68–75. doi: 10.1016/j.jpsychires.2018.11.018, PMID: 30508745 PMC6347462

[24] Subirà M Sato JR Alonso P do Rosário MC Segalàs C Batistuzzo MC . Brain structural correlates of sensory phenomena in patients with obsessive-compulsive disorder. J Psychiatry Neurosci. (2015) 40:232–40. doi: 10.1503/jpn.140118, PMID: 25652753 PMC4478056

[25] Horder J Wilson CE Mendez MA Murphy DG . Autistic traits and abnormal sensory experiences in adults. J Autism Dev Disord. (2014) 44:1461–9. doi: 10.1007/s10803-013-2012-7, PMID: 24305777 PMC4022987

[26] Rosario-Campos MC Leckman JF Mercadante MT Shavitt RG Prado HS Sada P . Adults with early-onset obsessive-compulsive disorder. Am J Psychiatry. (2001) 158:1899–903. doi: 10.1176/appi.ajp.158.11.1899, PMID: 11691698

[27] Kong XZ Boedhoe PSW Abe Y Alonso P Ameis SH Arnold PD . Mapping cortical and subcortical asymmetry in obsessive-compulsive disorder: findings from the ENIGMA consortium. Biol Psychiatry. (2020) 87:1022–34. doi: 10.1016/j.biopsych.2019.04.022, PMID: 31178097 PMC7094802

[28] Weeland CJ White T Vriend C Muetzel RL Starreveld J Hillegers MHJ . Brain morphology associated with obsessive-compulsive symptoms in 2,551 children from the general population. J Am Acad Child Adolesc Psychiatry. (2021) 60:470–8. doi: 10.1016/j.jaac.2020.03.012, PMID: 32949714

[29] First MB . Structured clinical interview for the DSM (SCID). In: Cautin RL Lilienfeld SO , editors. The Encyclopedia of Clinical Psychology. John Wiley & Sons, Hoboken, NJ (2015). p. 1–6.

[30] Kano Y Matsuda N Nonaka M Fujio M Kuwabara H Kono T . Sensory phenomena related to tics, obsessive-compulsive symptoms, and global functioning in Tourette syndrome. Compr Psychiatry. (2015) 62:141–6. doi: 10.1016/j.comppsych.2015.07.006, PMID: 26343478

[31] Goodman WK Price LH Rasmussen SA Mazure CM Fleischmann RL Hill CL . The Yale–Brown Obsessive Compulsive Scale. I. Development, use, and reliability. Arch Gen Psychiatry. (1989) 46:1006–11. doi: 10.1001/archpsyc.1989.01810110048007, PMID: 2684084

[32] Kuckertz JM Rhee DW Schreck M Van Kirk N Dattolico D Falkenstein MJ . Reevaluating the psychometric properties of symptom measures within longitudinal contexts: the Yale–Brown Obsessive Compulsive Scale and Dimensional obsessive-compulsive Scale. Psychol Assess. (2021) 33:756–65. doi: 10.1037/pas0001017, PMID: 33829846

[33] Baron-Cohen S Wheelwright S Skinner R Martin J Clubley E . The Autism-Spectrum Quotient (AQ): evidence from Asperger syndrome/high-functioning autism, males and females, scientists and mathematicians. J Autism Dev Disord. (2001) 31:5–17. doi: 10.1023/a:1005653411471, PMID: 11439754

[34] Wakabayashi A Tojo Y Baron-Cohen S Wheelwright S . The Autism-Spectrum Quotient (AQ) Japanese version: evidence from high-functioning clinical group and normal adults. Shinrigaku Kenkyu. (2004) 75:78–84. doi: 10.4992/jjpsy.75.78, PMID: 15724518

[35] Thompson E . Hamilton rating scale for anxiety (HAM-A). Occup Med (Lond). (2015) 65:601. doi: 10.1093/occmed/kqv054, PMID: 26370845

[36] Shear MK Vander Bilt J Rucci P Endicott J Lydiard B Otto MW . Reliability and validity of a structured interview guide for the Hamilton Anxiety Rating Scale (SIGH-A). Depress Anxiety. (2001) 13:166–78. doi: 10.1002/da.1033, PMID: 11413563

[37] Mimura C Nishioka M Sato N Hasegawa R Horikoshi R Watanabe Y . A Japanese scale to assess anxiety severity: development and psychometric evaluation. Int J Psychiatry Med. (2011) 41:29–45. doi: 10.2190/PM.41.1.d, PMID: 21495520

[38] Wigham S Rodgers J South M McConachie H Freeston M . The interplay between sensory processing abnormalities, intolerance of uncertainty, anxiety and restricted and repetitive behaviours in autism spectrum disorder. J Autism Dev Disord. (2015) 45:943–52. doi: 10.1007/s10803-014-2248-x, PMID: 25261248

[39] Prato A Saia F Ferrigno M Finocchiaro V Barone R Rizzo R . Sensory phenomena in children with Tourette syndrome or autism spectrum disorder. Front Psychiatry. (2024) 15:1338234. doi: 10.3389/fpsyt.2024.1338234, PMID: 38628261 PMC11018939

[40] Shaw P Kabani NJ Lerch JP Eckstrand K Lenroot R Gogtay N . Neurodevelopmental trajectories of the human cerebral cortex. J Neurosci. (2008) 28:3586–94. doi: 10.1523/JNEUROSCI.5309-07.2008, PMID: 18385317 PMC6671079

[41] McDougle CJ Goodman WK Leckman JF Lee NC Heninger GR Price LH . Haloperidol addition in fluvoxamine-refractory obsessive-compulsive disorder. A double-blind, placebo-controlled study in patients with and without tics. Arch Gen Psychiatry. (1994) 51:302–8. doi: 10.1001/archpsyc.1994.03950040046006, PMID: 8161290

[42] Bloch MH Landeros-Weisenberger A Kelmendi B Coric V Bracken MB Leckman JF . A systematic review: antipsychotic augmentation with treatment refractory obsessive-compulsive disorder. Mol Psychiatry. (2006) 11:622–32. doi: 10.1038/sj.mp.4001823, PMID: 16585942

[43] Robertson AE Simmons DR . The relationship between sensory sensitivity and autistic traits in the general population. J Autism Dev Disord. (2013) 43:775–84. doi: 10.1007/s10803-012-1608-7, PMID: 22832890

