# Supplementary material for: Earlier age at onset is associated with more severe sensory phenomena in drug-naive, comorbidity-free patients with obsessive-compulsive disorder
Source: Front Psychiatry. 2026 Mar 11;17:1774594. doi: 10.3389/fpsyt.2026.1774594 (PMC13034176; doi:10.3389/fpsyt.2026.1774594)
Supplement: Supplementary file 1 [file Table1.docx]

**Supplementary Materials**

Supplementary Table S1. Comorbid psychiatric disorders in excluded patients with OCD [Number]

| **Comorbid Disorder Category** | **Specific Diagnosis** | **n** |
| --- | --- | --- |
| **Anxiety Disorders** |  | 14 |
|  | Generalized Anxiety Disorder | 4 |
|  | Specific Phobia | 4 |
|  | Panic Disorder | 3 |
|  | Social Anxiety Disorder | 2 |
|  | Agoraphobia | 1 |
| **Mood Disorders** |  | 13 |
|  | Major Depressive Disorder | 12 |
|  | Bipolar Disorder | 1 |
| **Neurodevelopmental Disorders** |  | 6 |
|  | Tic Disorders | 4 |
|  | ADHD | 2 |
| **Obsessive-Compulsive and Related Disorders** |  | 3 |
|  | Hoarding Disorder | 2 |
|  | Body Dysmorphic Disorder | 1 |
| **Somatic Symptom and Related Disorders** | Not specified | 4 |
| **Feeding and Eating Disorders** | Not specified | 2 |
| **Substance-Related and Addictive Disorders** |  | 2 |
|  | Alcohol Use Disorder | 1 |
|  | Other (non-alcohol) substance use | 1 |
| **Trauma- and Stressor-Related Disorders** | PTSD | 1 |

Values represent the number of diagnoses. Because participants could meet criteria for more than one comorbid disorder, counts across rows may exceed the number of excluded participants with comorbidity. Abbreviations: OCD, Obsessive-Compulsive Disorder; ADHD, Attention-Deficit/Hyperactivity Disorder; PTSD, Post-Traumatic Stress Disorder.

Supplementary Table S2. Lifetime psychotropic medication history among patients excluded due to psychotropic medication use (n = 47) [Number of patients]

| **Medication Class** | **Specific Type** | **n** |
| --- | --- | --- |
| **Antidepressants** |  | 45 |
|  | Selective Serotonin Reuptake Inhibitors | 39 |
|  | Tricyclic Antidepressants | 4 |
|  | Tetracyclic Antidepressants | 1 |
|  | Serotonin-Norepinephrine Reuptake Inhibitors | 1 |
| **Antipsychotics** |  | 11 |
|  | Aripiprazole | 9 |
|  | Olanzapine | 1 |
|  | Risperidone | 1 |
| **Benzodiazepines** |  | 7 |
|  | Alprazolam | 1 |
|  | Bromazepam | 3 |
|  | Brotizolam | 1 |
|  | Ethyl loflazepate | 1 |
|  | Etizolam | 1 |
| **Non-benzodiazepine hypnotics** | Zolpidem | 1 |
| **Mood Stabilizers** |  | 2 |
|  | Lithium | 1 |
|  | Valproate | 1 |

Values represent the number of patients with a lifetime history of each medication class/type. Because patients could have taken more than one medication class/type, counts across medication classes may sum to more than the number of excluded patients with medication history.

Supplementary Table S3. Two-part analyses addressing the floor effect at USP-SPS = 0 (past month)

Panel A. Part 1: Logistic regression predicting current SP (USP-SPS > 0 vs USP-SPS = 0) (N = 30)

| **Predictor** | **OR** | **95% CI** | ***p*-value** |
| --- | --- | --- | --- |
| Age at onset | 0.93 | 0.84–1.01 | 0.076 |
| Sex (female vs male) | 1.52 | 0.05–31.70 | 0.779 |
| AQ total | 1.24 | 1.04–1.57 | 0.012* |
| Y-BOCS total | 0.84 | 0.62–1.04 | 0.109 |

Panel B. Part 2: Linear regression predicting USP-SPS severity among participants with USP-SPS > 0 (n = 23)

| **Predictor** | ***B* (unstandardized)** | ***SE*** | ***t*** | ***p*-value** |
| --- | --- | --- | --- | --- |
| Age at onset (years) | −0.144 | 0.068 | −2.10 | 0.0497* |
| Sex (female vs male) | 1.149 | 0.807 | 1.42 | 0.172 |
| AQ total | 0.114 | 0.137 | 0.83 | 0.417 |
| Y-BOCS total | −0.030 | 0.140 | −0.22 | 0.831 |

Panel C. Part 2: Linear regression excluding one influential observation (n = 22)

| **Predictor** | ***B* (unstandardized)** | ***SE*** | ***t*** | ***p*-value** |
| --- | --- | --- | --- | --- |
| Age at onset (years) | −0.134 | 0.056 | −2.37 | 0.030* |
| Sex (female vs male) | 1.288 | 0.666 | 1.94 | 0.070 |
| AQ total | 0.029 | 0.116 | 0.25 | 0.803 |
| Y-BOCS total | 0.127 | 0.126 | 1.01 | 0.328 |

In Panel A, the dependent variable was the presence of current SP (USP-SPS > 0 vs = 0). Model fit for Panel A: likelihood ratio *χ²*(4) = 11.91, *p* = 0.018. Odds ratios (ORs) are reported per 1-unit increase in continuous predictors; 95% CIs are Wald-based. In Panels B and C, the dependent variable was USP-SPS severity among participants with USP-SPS > 0. Model fit: Panel B adjusted *R²* = 0.122, *F*(4,18) = 1.76, *p* = 0.180; Panel C adjusted *R²* = 0.327, *F*(4,17) = 3.55, *p* = 0.028. Sex was entered as a categorical variable. Abbreviations: USP-SPS, University of São Paulo Sensory Phenomena Scale; SP, sensory phenomena; AQ, Autism-Spectrum Quotient; Y-BOCS, Yale-Brown Obsessive Compulsive Scale; SE, standard error. **p* < 0.05.
